# Cryptic Genetic Diversity within the *Anopheles nili* group of Malaria Vectors in the Equatorial Forest Area of Cameroon (Central Africa)

**DOI:** 10.1371/journal.pone.0058862

**Published:** 2013-03-14

**Authors:** Cyrille Ndo, Frédéric Simard, Pierre Kengne, Parfait Awono-Ambene, Isabelle Morlais, Igor Sharakhov, Didier Fontenille, Christophe Antonio-Nkondjio

**Affiliations:** 1 Laboratoire de Recherche sur le Paludisme, Organisation de Coordination pour la lutte Contre les Endémies en Afrique Centrale, Yaoundé, Cameroon; 2 Unité de Recherche Mixte Maladies Infectieuses et Vecteurs : Ecologie, Génétique, Evolution et Contrôle, Institut de Recherche pour le Développement, Montpellier, France; 3 Faculty of Medicine and Pharmaceutical Sciences, University of Douala, Douala, Cameroon; 4 Department of Entomology, Virginia Tech, Blacksburg, Virginia, United States of America; 5 Faculty of Health Sciences, University of Bamenda, Bambili, Cameroon; Kansas State University, United States of America

## Abstract

**Background:**

The *Anopheles nili* group of mosquitoes includes important vectors of human malaria in equatorial forest and humid savannah regions of sub-Saharan Africa. However, it remains largely understudied, and data on its populations’ bionomics and genetic structure are crucially lacking. Here, we used a combination of nuclear (i.e. microsatellite and ribosomal DNA) and mitochondrial DNA markers to explore and compare the level of genetic polymorphism and divergence among populations and species of the group in the savannah and forested areas of Cameroon, Central Africa.

**Principal Findings:**

All the markers provided support for the current classification within the *An. nili* group. However, they revealed high genetic heterogeneity within *An. nili s.s.* in deep equatorial forest environment. Nuclear markers showed the species to be composed of five highly divergent genetic lineages that differed by 1.8 to 12.9% of their Internal Transcribed Spacer 2 (ITS2) sequences, implying approximate divergence time of 0.82 to 5.86 million years. However, mitochondrial data only detected three major subdivisions, suggesting different evolutionary histories of the markers.

**Conclusions/Significance:**

This study enlightened additional cryptic genetic diversity within *An. nili s.s.* in the deep equatorial forest environment of South Cameroon, reflecting a complex demographic history for this major vector of malaria in this environment. These preliminary results should be complemented by further studies which will shed light on the distribution, epidemiological importance and evolutionary history of this species group in the African rainforest, providing opportunities for in-depth comparative studies of local adaptation and speciation in major African malaria vectors.

## Introduction

In the equatorial forest regions of Africa, malaria remains endemic despite significant efforts in treatment of the disease and vector control [1]. Part of explanation for this situation resides in the fact that a number of genetically, behaviorally and ecologically distinct species and populations of *Anopheles* mosquitoes can and do transmit *Plasmodium*, the malignant agent of the disease, simultaneously or replace each other seasonally insuring year-round transmission [2]. Unfortunately, many of these vectors might escape traditional vector control operations by Indoor Residual Spraying of insecticides (IRS) and Insecticide-Treated bed Nets (ITNs), which are directed against indoor biting and/or resting mosquitoes [3], [4]. This is exemplified in the *Anopheles nili* group of malaria vectors.

The *An. nili* group includes four closely related species namely *An. nili sensu stricto* (thereafter, *An. nili s.s.*), *An. somalicus*, *An. carnevalei* and *An. ovengensis* which can be identified through slight morphological diagnostic characters present at the larval and/or adult stages [5]–[7], and by a species-specific PCR assay [8]. Their larvae typically develop in river networks with a marked preference for the edges of fast running streams and rivers exposed to light and containing vegetation and/or floating debris [9]. *Anopheles nili s.s.* is widespread and is found throughout sub-Saharan Africa. It is highly anthropophilic, biting man and resting indoors as well as outdoors [5], [10]–[16]. The other members of the group are more localized within the equatorial forest region of Central Africa, although their true distribution range remains largely unknown [9], [17], [18]. *Anopheles somalicus* is not involved in malaria transmission because of its preference for non-human blood and exophilic habits [2], [19]. A comprehensive study in Cameroon confirmed that *An. nili s.s.* is the major malaria vector of the group and emphasized the exophagic behavior of *An. ovengensis* and *An. carnevalei* [20]. In Equatorial Guinea, sporozoïte rates in *An. ovengensis* can reach 4.1% (n = 74), which is higher than that of *An. gambiae* in the same area (3.3%, n = 603, [17]), confirming its major role in the epidemiology of malaria. Moreover, recent studies found the circulation of various *Plasmodium* species and lineages related to *P. falciparum* in great apes and infection of gorillas and guenons by *P. falciparum* in the central forest region of Cameroon and Gabon [21]–[25]. However, the potential mosquito vectors have not been identified yet. These discoveries further bring urgency to the study of the taxonomic status of members of the *An. nili* group and their possible role in transmission of various *Plasmodium* species.

A recent study using 11 microsatellite markers demonstrated a significant genetic differentiation of the forest *An. nili s.s.* population of Kenge in the Democratic Republic of Congo (DRC) when compared to other populations from humid savannah of Central and West Africa [18]. Both local adaptation and geographic isolation could cause this differentiation. Extensive allele sharing between populations and homogeneity across loci suggested that enhanced genetic drift rather than selection was responsible for the observed pattern. Fluorescence *in situ* hybridization (FISH) mapping of the microsatellite loci on a recently developed cytogenetic map for *An. nili* [26], [27] demonstrated physical independence of the markers, strengthening the view that enhanced genetic drift, rather than selection, was responsible for reduced variability and increased differentiation of the Kenge population. These data strongly suggest existence of ecological and geographical barriers that affect gene flow among *An. nili* natural populations in equatorial Africa.

Here, we used nuclear (i.e. microsatellites and rDNA) and mitochondrial DNA markers to further explore the genetic structure of members of the *An. nili* group in South Cameroon, where all four members of the group are known to occur [7], [8], [20]. We document further cryptic genetic substructure within *An. nili s.s.*, reflecting a complex demographic history. We advocate for further evaluation of the impact of this genetic heterogeneity and its relevance for malaria epidemiology and control.

## Materials and Methods

### Ethics Statement

This study received the approval of the National Ethics Committee of Cameroon (Ref: FWA IRB0001954). No other specific permits were required. No location was privately-owned or protected and field studies did not involve endangered or protected species. Consent was obtained from all volunteers participating in the study, and they were monitored for signs of fever and treated for all diagnosed malaria cases.

### Mosquito Collection and Identification

**Figure 1:**
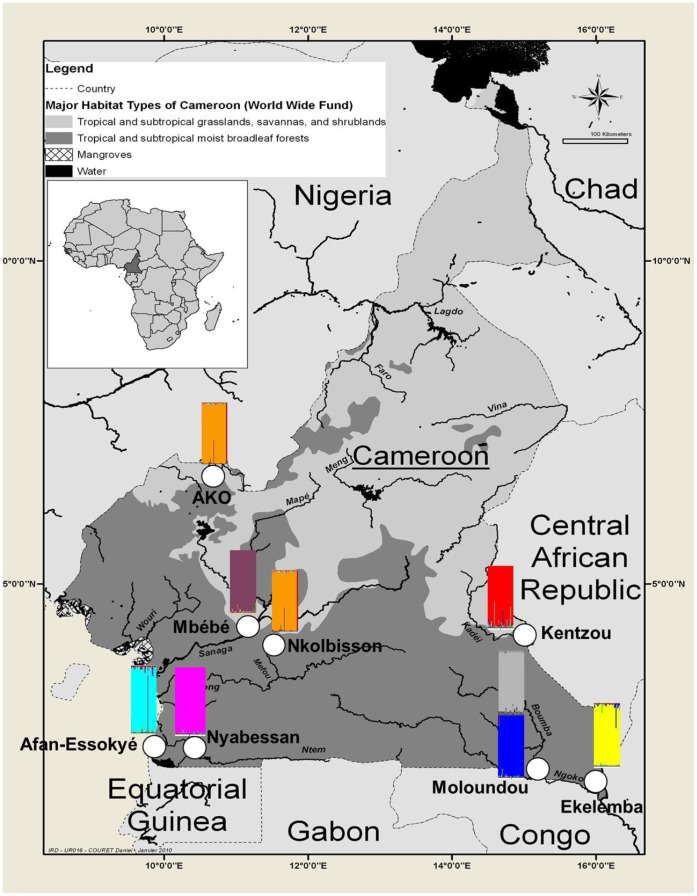
Map of Cameroon showing collection sites and the 8 microsatellites clusters detected by Bayesian clustering analysis using STRUCTURE. Each colored rectangle represents a distinct genetic cluster within which each vertical bar corresponds to a single individual. Note that Ako and Nkolbisson specimens belong to the same genetic cluster (see text).

Mosquitoes were collected from 2007 to 2009 in eight riverside villages situated in different ecological settings in Cameroon including Afan-Essokyé, Ako, Ekelemba, Kentzou, Moloundou, Mbébé, Nkolbisson, and Nyabessan (Figure 1, Table 1). Field-collected larvae and adult *An. nili* s.l. mosquitoes were first sorted using morphological diagnostic characters [5]–[7], [28]. Adult females were stored individually in numbered tubes containing a desiccant whereas larvae were preserved in tubes containing 95% ethanol. Then, genomic DNA was extracted using a standard protocol [29] and morphological identification was confirmed by PCR [8].

**Table 1:** Characteristics of sampling sites and species collection methods and dates.

Sites	Geographicalcoordinates	Hydrographicnetwork	Ecologicaldomain^b^	Species^a^	Stage	Method ofCollection^c^	Collection date
Ako	6°49′N, 10°43′E	Atoro and Akon	HS	*An. nili s.s.*	Adult	HLC	03/2007
Nkolbisson	3°52′N; 11°25′E	Mefou	DF	*An. nili s.s.*	Adult	PSC	04/2008
Kentzou	4°09′N, 15°02′E	Kadei	DF	*An. nili s.s.*	Larva	LC	02/2009
Moloundou	2°02′N, 15°12′E	Boumba and Ngoko	DEF	*An. nili s.s.*	Larva+Adult	HLC/LC	10/2008 & 02/2009
Ekelemba	1°49′N, 15°54′E	Baad	DEF	*An. nili s.s.*	Larva	LC	02/2009
Mbébé	4°10′N; 11°04′E	Sanaga	DF	*An. somalicus*	Larva	LC	03/2009
Nyabessan	2°10′N; 10° 40′E	Ntem and Njoh	DEF	*An. ovengensis*	Adult	HLC	12/2007
Afan-Essokyé	2°22′N; 9°59′E	Bitandé	DEF	*An. carnevalei*	Adult	HLC	12/2007

a morphological and molecular identification;

b HS, Humid Savannah; DF, Degraded forest; DEF, Deep evergreen forest;

c HLC, Human Landing Collection; PSC, Pyrethrum Spray Catch; LC, Larval Collection through the dipping technique [67].

### Microsatellite Genotyping and Analysis

Eleven microsatellite loci developed in *An. nili s.s.* [27], [30] were used to amplify *An. carnevalei*, *An. ovengensis* and *An. somalicus* DNA. Genotypes were determined for individual mosquitoes as previously described [18]. Genetic diversity at each microsatellite locus within each sample was characterized by estimates of allelic richness (Rs) [31] and unbiased expected heterozygosity (He) under Hardy-Weinberg Equilibrium (HWE), using FSAT V2.9.3.2 [32]. This program was also used to test for conformance of genotype frequencies to HWE for each locus and each sample, and to explore Linkage Disequilibrium (LD) between microsatellite loci. The frequency of null alleles at each locus within each population was determined using GENEPOP V4.0 [33], [34], and the allele and genotype frequencies were then adjusted accordingly in MICROCHECKER V2.2.3 [35]. The null allele adjusted dataset was compared to the original dataset to explore the effect of null alleles on estimates of genetic differentiation.

Heterozygosity tests were implemented in BOTTLENECK V1.0.02 [36] in order to assess deviation from Mutation-Drift Equilibrium (MDE). The tests were performed using the Stepwise Mutation Model (SMM) and the Two Phase Model (TPM), with 10–30% indels larger than one repeat unit. Statistical significance of the tests was assessed for each sample across all loci by the Wilcoxon signed-rank test available in BOTTLENECK.

Bayesian clustering analysis was implemented in STRUCTURE V2.2 [37] to estimate the most probable number of discrete populations or genetic clusters (K) in the dataset, while minimizing Hardy-Weinberg and gametic phase disequilibrium between loci within clusters, with no a priori assumptions of population structure. The admixture model, which assumes that a fraction of the genome of each individual can come from one of each population, was used. Allele frequencies were allowed to be correlated assuming that the populations diverged from a single ancestral population at some point in the past, and the differences in their allele frequencies are the result of drift that has occurred since their divergence [37]. The method of Evanno *et al.* [38] was used to determine the most likely number of clusters. Differentiation among clusters was examined with Wright F-statistics [39] computed according to Weir and Cockerham [40] using FSTAT. For the calculation of *Fst*, monomorphic loci were excluded from the analysis and significance tests were conducted using a randomization approach as implemented in FSTAT.

The number of putative migrants (*Nm*) per generation between populations was estimated from pairwise *Fst* [41]. The contribution of geographical distance to the pattern of differentiation observed was investigated by the regression of *Fst/*(1-*Fst*) on geographic distance, assuming migration in one direction (i.e. along hydrographic networks) [42]. The significance of this regression was tested using a mantel test available in GENEPOP [43].The Bonferroni correction [44] was used to adjust the nominal significance level whenever multiple tests were performed.

### DNA Sequencing and Phylogenetic Analysis

Sequencing was performed using the primers set and protocol described in Ndo *et al.* [18]. Ten specimens per geographical sample (but twenty from Moloundou) among those used for microsatellite analysis were sequenced for the Internal Transcribed Spacer 2 (ITS2) and domain-3 (D3) of nuclear 28S ribosomal DNA, and for *Cytochrome Oxydase subunit II* (*COII*) and *NADH deshydrogenase subunit IV* (*ND4*) of mitochondrial DNA.

Basic sequence polymorphism statistics and various population parameters were computed using DnaSP V5.10.01 [45] and MEGA V5.05 [46]. Genetic diversity was measured by assessing haplotype diversity (hd) and nucleotide diversity (π). MEGA was also used to compute genetic distances between haplotypes and/or populations [47]. The rate of the ITS2 clock of 2.2% per million years (myr), as assessed within the *Drosophila melanogaster* and *D. virilis* groups [48], was used to estimate divergence time between sequences. For mtDNA sequences, the conventional mtDNA rate of 2% per myr was used [49].

The Neighbour-Joining (NJ) method [50] performed in MEGA was used for the construction of phylogenetic trees, which were rooted using *An. moucheti* sequences ([63]; Ndo, unpublished) as an outgroup.

## Results

### Samples Composition

All mosquitoes from Ako, Ekelemba, Kentzou, Moloundou and Nkolbisson were morphologically and molecularly identified as *An. nili s.s.*, although some specimens from Moloundou and Ekelemba failed to amplify even after repeated attempts, possibly due to poor DNA quality. All individuals from Nyabessan, Afan-Essokyé and Mbébé were *An. ovengensis*, *An. carnevalei* and *An. somalicus* respectively, but *An. nili s.s* is also known to be present in the two later localities [9]. We never collected *An. somalicus* on human baits owing to its zoophilic behavior. Similarly, attempts to collect *An. nili s.s.* on human baits in Kentzou were unsuccessful suggesting possible zoophilic behavior of the species in this locality.

### Microsatellite Analysis

#### Genetic variability

A total of 363 individual mosquitoes were genotyped and there were marked differences in amplification profiles and polymorphism of loci among species, as well as between *An. nili s.s.* populations (Table 2). All 11 loci readily amplified and were polymorphic in *An. nili s.s.* samples from Ako and Nkolbisson, whereas only 10 (90.9%) successfully amplified in Kentzou (9 polymorphic) and Moloundou (9 polymorphic), falling down to 8 (73%) in Ekelemba (with only 5 loci being polymorphic). Similarly, only 9 (82%) loci successfully amplified *An. carnevalei* and *An. ovengensis* DNA, among which 7 and 6 respectively were polymorphic. In *An. somalicus*, 8 (73%) loci were scored successfully, with only 5 showing more than one allele (Table 2). These differences in locus amplification and polymorphism resulted in high genetic variability within the *An. nili s.s.* populations from Ako and Nkolbisson (Rs: 8.33–8.93, He: 0.833–0.857), whereas polymorphism was much lower in the other samples (Rs: 1.77–6.46, He: 0.231–0.659).

**Table 2:** Genetic variability at eleven microsatellite loci in 8 *An. nili* s.l. populations from Cameroon.

	*An. nili s.s.*					*An. carnevalei*	*An. ovengensis*	*An. somalicus*
Locus	Ako	Nkolbisson	Kentzou	Moloundou	Ekelemba	Afan- Essokyé	Nyabessan	Mbébé
	41	38	53	71	32	35	48	46
1D80								
*Rs*	10.81	8.27	5.99	10.50	3.50	6.04	7.82	5.11
*He*	0.917	0.869	0.713	0.913	0.310	0.775	0.829	0.765
*Fis*	0.151**	0.074	0.093	**0.232** ***	−0.115	0.052	**0.731** ***	0.148
1A27								
*Rs*	13.03	11.75	11.63	12.61	6.93	–	5.07	–
*He*	0.946	0.937	0.927	0.948	0.739	–	0.571	–
*Fis*	0.103*	**0.269** ***	0.091*	**0.239** ***	−**0.358** ***	–	0.203*	**–**
2Ateta								
*Rs*	9.22	8.92	7.54	5.11	1.31	7.36	6.73	3.82
*He*	0.861	0.886	0.849	0.696	0.030	0.817	0.784	0.610
*Fis*	0.011	0.112	0.095	**0.585** ***	0.000	0.044	0.036	0.144
A14								
*Rs*	8.60	6.06	1.19	1.30	1.00	1.00	1.00	5.83
*He*	0.858	0.802	0.019	0.083	0.000	0.000	0.000	0.787
*Fis*	0.112	**0.255** **	0.000	**0.830** ***	–	–	**–**	**0.501** ***
A154								
*Rs*	9.31	7.17	1.45	8.65	3.00	6.11	6.16	–
*He*	0.825	0.680	0.051	0.887	0.660	0.793	0.769	–
*Fis*	0.114*	0.002	1.000	**0.821** ***	−0.086	**0.678** ***	**0.501** ***	–
2C157								
*Rs*	5.92	4.84	1.00	1.00	1.00	2.00	1.00	1.00
*He*	0.766	0.670	0.000	0.000	0.000	0.507	0.000	0.000
*Fis*	−0.018	0.063	–	–	–	**1.000** ***	–	–
F56								
*Rs*	10.34	9.30	4.82	8.45	–	4.99	5.57	3.29
*He*	0.907	0.891	0.665	0.901	–	0.844	0.617	0.830
*Fis*	**0.360** ***	0.089	0.250**	**0.232** ***	–	**0.556** ***	0.027	0.199**
B115								
*Rs*	6.20	6.22	4.58	7.19	1.62	1.00	6.99	1.00
*He*	0.802	0.814	0.677	0.791	0.088	0.000	0.838	0.000
*Fis*	−0.143	−0.027	−0.117	**0.499** ***	−0.033	–	0.036	–
F41								
*Rs*	11.76	14.43	5.45	8.93	1.00	6.63	1.00	11.34
*He*	0.935	0.960	0.733	0.864	0.000	0.785	0.000	0.919
*Fis*	0.080	−0.005	−0.159*	**0.323** ***	–	0.187	**–**	0.064
1F43								
*Rs*	6.57	6.41	4.53	7.32	–	–	–	1.00
*He*	0.817	0.804	0.400	0.508	–	–	–	0.000
*Fis*	**0.320** ***	−0.048	0.116	**0.641** ***	–	–	**–**	–
1G13								
*Rs*	6.51	8.30	–	–	–	6.84	–	–
*He*	0.783	0.851	–	–	–	0.692	–	–
*Fis*	0.009	**0.254** ***	–	–	–	0.281**	–	–
Mean over loci								
*Rs*	8.93	8.33	4.38	6.46	1.77	3.81	3.76	3.29
*He*	0.857	0.833	0.503	0.659	0.231	0.579	0.490	0.489
*Fis*	0.104	**0.098** ***	0.060*	**0.437** ***	0.060	**0.374** ***	**0.269** ***	**0.210** ***

* P<0.05;

** P<0.01;

*** P<0.001; Bold characters denote a significant heterozygote deficiency after correction for multiple testing by the sequential Bonferroni procedure;

*He*: expected heterozygosity; *Rs*: allelic richness calculated based on minimum size of 10 individuals per population [31] -: no amplification or no polymorphism detected; *Fis* are estimated following Weir & Cockerham [40].

### Hardy-Weinberg Equilibrium and Linkage Disequilibrium

Only polymorphic loci were considered for HWE and LD analyses. A total of 21 (33.33%) tests out of 63 were found out of HWE (P<0.001) after Bonferroni correction for multiple testing (Table 2). Within *An. nili s.s.,* the sample from Moloundou had the highest number of deviations. In fact, all 9 polymorphic loci were out of HWE, associated with positive *Fis* values suggesting population substructure, although null alleles could also account for some deviation. By contrast, no more than 2 significant deviations were observed in all other *An. nili s.s.* samples. The number of significant deviations from HWE detected in the other members of the group varied from 1 (out of 5 polymorphic loci) in *An. somalicus* to 3 (out of 7 polymorphic loci) in *An. carnevalei* (Table 2). The frequency of null alleles at those loci showing significant deviation from HWE ranged from 8.73 to 41.65% (Table S1), suggesting mutations in the primer-annealing sequences were probably involved. Nevertheless, nulls alleles did not significantly bias our subsequent interpretation of populations’ differentiation.

Exact tests for LD within each *An. nili s.s.* population resulted in 5 (2.6%) significant values (P<0.05) out of 192 comparisons after correction by the Bonferroni procedure. All these deviations were detected in Moloundou where all polymorphic loci were found out of HWE, and involved loci 1D80 and F41, 2Ateta and F41, B115 and F41, 2Ateta and 1A27, and B115 and F56. Because all these loci are located on different chromosomal arms [27], this result further supports HWE analysis in pointing towards splitting of the gene pool in the Moloundou population (i.e. the Wahlund’s effect). No significant LD between loci was detected in *An. carnevalei*, *An. ovengensis* and *An. somalicus* populations.

### Genetic Differentiation and Gene Flow

The Bayesian clustering analysis using the software STRUCTURE was implemented in the expectation that the genotypes would segregate into four main clusters corresponding to the four previously recognized species within the *An. nili* group. The optimal number of clusters detected based on the method of Evano *et al.* [38] was K = 8 (Figure 1). *Anopheles carnevalei*, *An. somalicus* and *An. ovengensis* samples each formed a distinct and homogeneous genetic cluster, while five clusters were defined within *An. nili s.s.* Specimens sampled in savannah (Ako) and degraded forest (Nkolbisson) areas were merged together into a single genetic cluster, whereas four clusters were detected among populations sampled within the evergreen forest block: one in Kentzou, one in Ekelemba and two in Moloundou, namely ‘Moloundou A’ and ‘Moloundou B’ in subsequent analyses.

High levels of genetic differentiation were recorded between species. The overall *Fst* estimate across all loci was 0.373 and was highly significant (P<0.0001). Mean pairwise *Fst* over all loci varied from 0.201 to 0.659 (P<0.0001) with corresponding *Nm* values in the range of 0.14 to 1.16, suggesting very restricted gene flow between species (Table 3).

**Table 3:** Pairwise microsatellite *Fst* (below) and corresponding *Nm* (above) values between *An. nili* s.l. populations from Cameroon.

	*An. nili s.s.*						*An. carnevalei*	*An. ovengensis*	*An. somalicus*
	Ako	Nkolbisson	Kentzou	Moloundou A	Moloundou B	Ekelemba	Afan-Essokyé	Nyabessan	Mbébé
Ako	–	23.97	0.56	0.88	0.50	0.35	0.74	0.55	0.54
Nkolbisson	0.011***	–	0.51	0.78	0.48	0.33	0.67	0.49	0.51
Kentzou	**0.303** ***	**0.313** ***	–	1.07	0.40	0.28	0.28	0.55	0.26
Moloundou A	**0.221** ***	**0.236** ***	**0.160** ***	–	0.62	0.40	0.40	1.16	0.30
Moloundou B	**0.350** ***	**0.367** ***	**0.343** ***	**0.296** ***	–	0.40	0.23	0.48	0.20
Ekelemba	**0.418** ***	**0.435** ***	**0.450** ***	**0.397** ***	**0.416** ***	–	0.14	0.26	0.13
Afan-Essokyé	**0.274** ***	**0.295** ***	**0.483** ***	**0.423** ***	**0.580** ***	**0.646** ***	–	0.28	0.36
Nyabessan	**0.315** ***	**0.341** ***	**0.311** ***	**0.201** ***	**0.380** ***	**0.531** ***	**0.495** ***	–	0.26
Mbébé	**0.313** ***	**0.319** ***	**0.476** ***	**0.435** ***	**0.575** ***	**0.659** ***	**0.452** ***	**0.491** ***	–

*** P<0.001. Bold characters denote a significant heterozygote deficiency after correction for multiple testing by the sequential Bonferroni procedure.

Within *An. nili s.s*., high levels of genetic differentiation (*Fst:* 0.16–0.45; P<0.0001) and correspondingly low migration index (*Nm:* 0.28–1.07) were estimated between populations, except between Ako and Nkolbisson which belong to the same genetic cluster (Table 3).

No significant correlation was detected between genetic and geographic distances (R^2^ = 0.0178; P = 0.304) and there was no indication of recent population contraction in any of the populations tested (data not shown).

### DNA Sequencing Analysis

#### rDNA polymorphism and divergence

The D3 domain of the 28S rDNA was amplified in 90 specimens (10 from each geographic population, and 20 from Moloundou) and the alignment length, including insertions/deletions (indels), was 382 bp (Figure S1). No sequence polymorphism (*π* = 0) was detected within each geographical population, except in Moloundou where two highly divergent sequence variants were observed (*π* = 0.034), corresponding to the Moloundou A and Moloundou B microsatellites clusters. Haplotypes detected in *An. carnevalei*, *An. ovengensis* and *An. somalicus* populations, and in *An. nili s.s.* from Ako and Nkolbisson were identical to those published by Kengne *et al.* [8] and Ndo et al. [18] (EMBL accession numbers: AJ429053 for *An. nili s.s*., AJ429052 for *An. ovengensis*, AJ438690 for *An. somalicus* and AJ429051 for *An. carnevalei*). The four remaining *An. nili s.s.* haplotypes were new and found exclusively in deep forest populations (GenBank accession numbers: KC189970– KC189973). They differed from one another, and from the deposited *An. nili s.s.* haplotype by 13 to 16 fixed nucleotide substitutions and many indels. Genetic distances between species’ haplotypes ranged from 0.3 to 5%. Similar genetic distances were also estimated between *An. nili s.s.* haplotypes, ranging from 0.5 to 5.9% (Table S2).

Likewise, there was no intra-population variation for the ITS2 region and the alignment length was 480 bp for *An. carnevalei*, 503 bp for *An. ovengensis*, 513 bp for *An. somalicus*, 451 bp for *An. nili s.s.* from Ako and Nkolbisson, 445 bp for *An. nili s.s.* from Kentzou and Moloundou A and 480 bp for sequences from Ekelemba and Moloundou B (Figure S2). ITS2 sequences also segregated into eight haplotypes, in straight correspondence with the 8 microsatellite clusters and the D3 sequence analysis. Four of these haplotypes were identical to those described earlier (EMBL accession numbers: AJ429048 for *An. nili s.s*., AJ429049 for *An. ovengensis*, AJ438689 for *An. somalicus* and AJ429050 for *An. carnevalei*) [8], [18]. The four new haplotypes were also observed in the deep forest *An. nili s.s.* populations (GenBank accession numbers: KC189966– KC189969). They differed from one another, and from the deposited *An. nili s.s.* haplotype by 30 to 95 nucleotide substitutions and many indels, suggesting high genetic divergence (Figure S2). Genetic distances between species were high, ranging from 5.8 to 18.6% which correspond to divergence times between 2.63 and 8.45 myr assuming a divergence rate of 2.2% per myr (above). Within *An. nili s.s.*, genetic distances between haplotypes ranged from 1.8 to 12.9%, implying approximate divergence time of 0.82 to 5.86 myr (Table S2).

**Figure 2:**
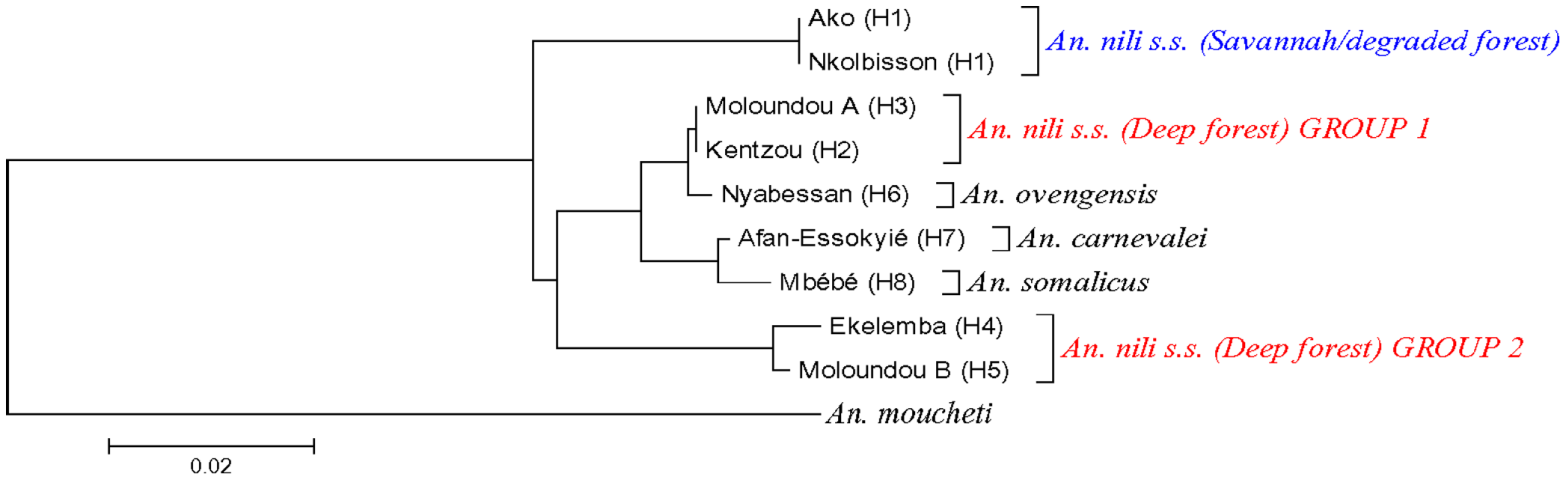
Neighbor-Joining (NJ) trees of *An. nili* s.l. D3 haplotypes from Cameroon. H1….Hn: Haplotype 1 to Haplotype n.

**Figure 3:**
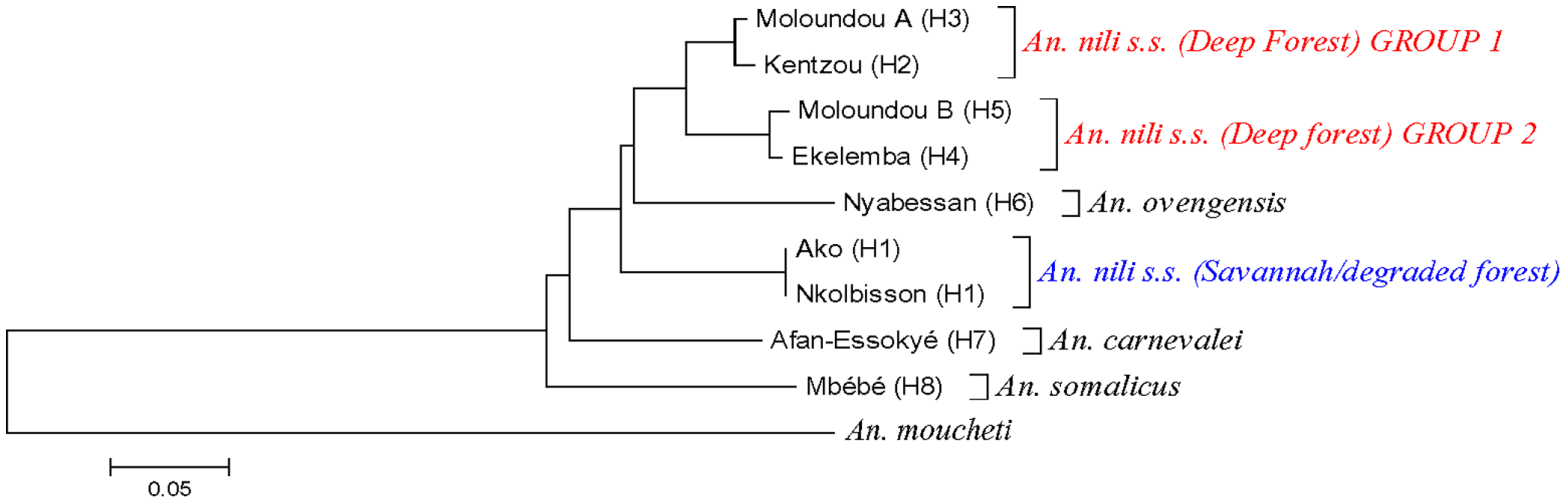
Neighbor-Joining (NJ) trees of *An. nili* s.l. ITS2 haplotypes from Cameroon. H1….Hn: Haplotype 1 to Haplotype n.

The topologies of the D3 and ITS2 phylogenetic trees were congruent, showing *An. nili* group to be monophyletic although relationship between species changed according to the marker. All the trees revealed three major groups among *Anopheles nili s.s.* specimens analyzed: 1) *An. nili s.s.* from savannah/degraded forest (Ako and Nkolbisson), 2) *An. nili s.s.* from deep forest group 1 (Kentzou and Moloundou A) and 3) *An. nili s.s.* from deep forest group 2 (Ekelemba and Moloundou B) (Figures 2 and 3). Interestingly, the two forest clusters appeared more closely related to *An. ovengensis* and other members of the group, than to *An. nili s.s.* from savannah/degraded forest areas.

### Mitochondrial DNA Polymorphism and Divergence

Mitochondrial DNA sequences were determined for a minimum of 81 individual mosquitoes and summary statistics for genetic polymorphism are given in Table 4. Alignment length was 603 bp and 319 bp for the *COII* and *ND4*, respectively. Variations among sequences only included base substitutions. Absence of indels and heterozygous sites therefore suggests that there was no nuclear transposed copy.

**Table 4:** Summary statistics for *COII* and *ND4* polymorphism in *An. nili* s.l. populations from Cameroon.

	*An. nili s.s.*						*An. carnevalei*	*An. ovengensis*	*An. somalicus*
	Ako	Nkolbisson	Kentzou	Moloundou A	Moloundou B	Ekelemba	Afan-Essokyé	Nyabessan	Mbébé
*COII*									
n	9	9	10	10	9	10	7	8	10
ns	603	603	603	603	603	603	603	603	603
s	0	0	6	3	6	1	12	3	7
h	1	1	5	3	2	2	5	4	6
dh	0	0	0.667	0.378	0.500	0.533	0.857	0.786	0.867
π	0	0	0.002	0.001	0.005	0.001	0.011	0.002	0.003
*k*	0	0	1.200	0.600	3.000	0.533	6.000	1.000	1.911
*D_T_*	nc	nc	−1.796*	−1.562	1.566	1.302	1.225	−0.431	−0.964
*D*	nc	nc	−2.081*	−1.784	1.372	0.804	0.747	−0.622	−1.131
*F*	nc	nc	−2.256*	−1.934	1.580	1.026	0.938	−0.642	−1.224
*Fs*	nc	nc	−1.803*	−0.459	5.000	1.029	0.648	−1.164	−1.953*
*ND4*									
*n*	8	8	10	10	8	10	7	8	10
*ns*	319	319	319	319	319	319	319	319	319
*s*	0	0	5	3	9	1	2	17	3
*h*	1	1	5	4	5	2	3	4	5
*hd*	0	0	0.822	0.533	0.857	0.200	0.524	0.750	0.822
*π*	0	0	0.005	0.002	0.016	0.001	0.002	0.020	0.003
*k*	0	0	1.489	0.600	5.035	0.200	0.571	7.929	1.022
*DT*	nc	nc	−0.631	−1.562	1.513	−1.112	−1.237	1.079	−1.063
*D*	nc	nc	−0.686	−1.784	1.161	−1.243	−1.296	1.571*	−0.338
*F*	nc	nc	−0.754	−1.933	1.378	−1.347	−1.374	1.619*	−0.577
*Fs*	nc	nc	−1.284	−1.964	0.807	−0.339	−0.922	3.474	−2.20**

nc: not calculated. n: number of sequences; s: number of polymorphic sites; h: number of haplotypes; hd: haplotype diversity; π : nucleotide diversity. *DT*: Tajima’s D; *D*: Fu and Li’s D; *F*: Fu and Li’s F; *Fs*: Fu’s Fs statistics. *P<0.05, **P<0.01 and ***P<0.001.

**Figure 4:**
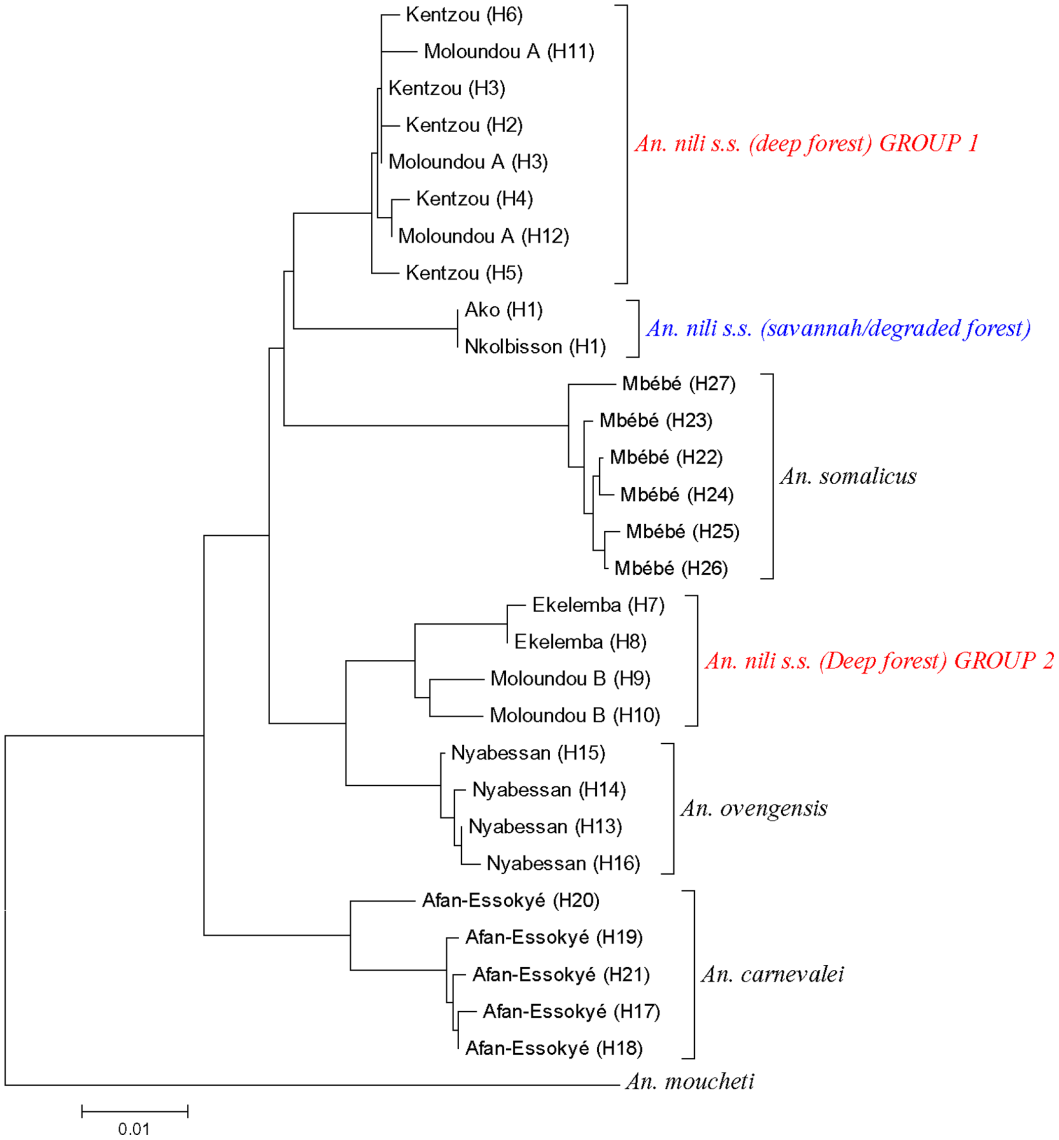
Neighbor-Joining (NJ) trees of *An. nili* s.l. *COII* haplotypes from Cameroon. H1….Hn: Haplotype 1 to Haplotype n.

**Figure 5:**
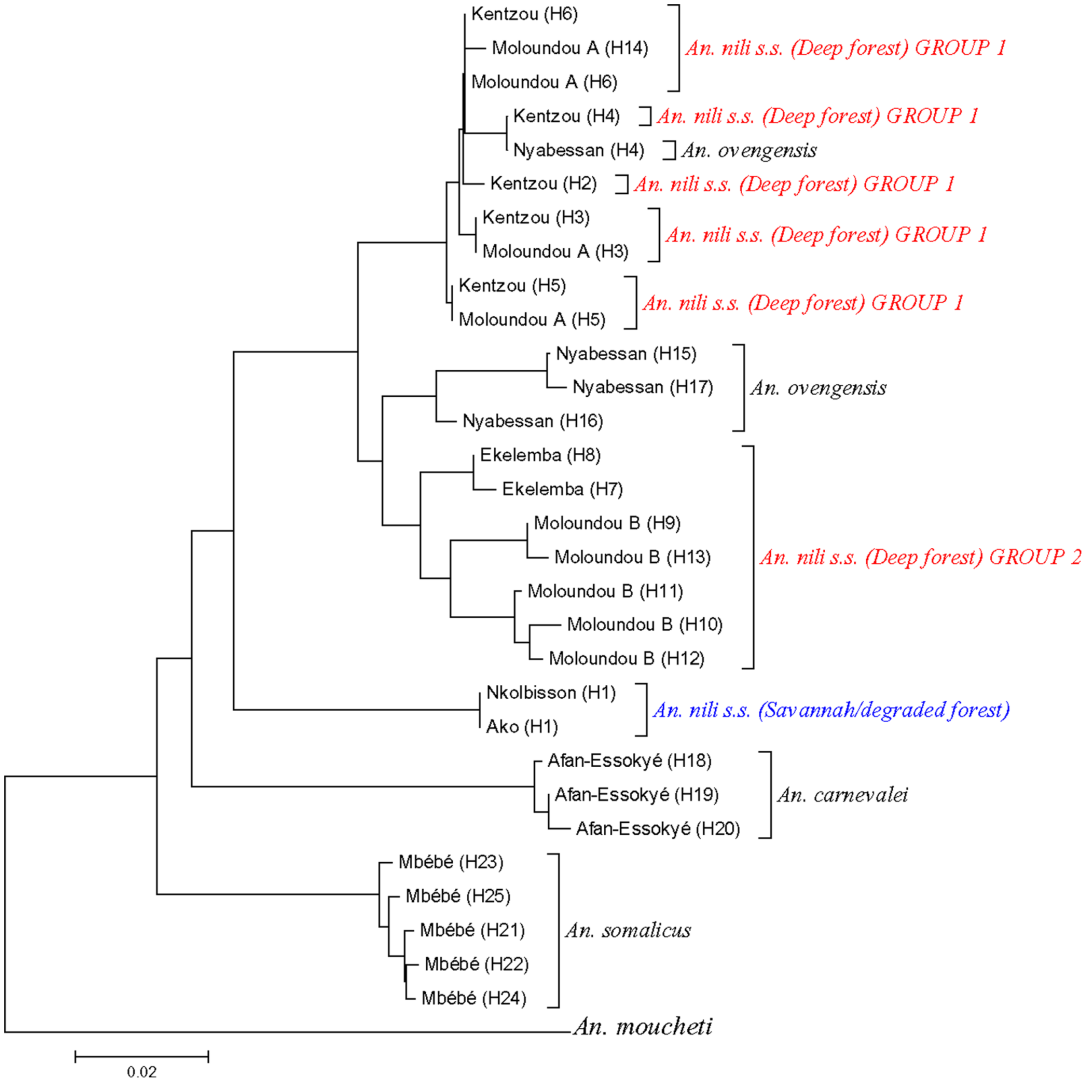
Neighbor-Joining (NJ) trees of *An. nili* s.l. *ND4* haplotypes from Cameroon. H1….Hn: Haplotype 1 to Haplotype n.

In contrast to rDNA data, there was no strong support for 8 distinct genetic clusters, although the existence of distinct genetic lineages within *An. nili s.s.* populations was confirmed. All the phylogenetic trees were congruent, showing *An. nili* group as monophyletic, with six clades: 1-*An. carnevalei*, 2-*An. ovengensis*, 3-*An. somalicus*, 4-*An. nili s.s.* from Ako and Nkolbssion 5-*An. nili s.s.* from Kentzou and Mouloundou A (deep forest group 1), and 6-*An. nili s.s.* from Ekelemba and Moloundou B (deep forest group 2) (Figures 4 and 5). However, the relationship between these clades was not clear as their position changed according to the gene tree. As observed above, *An. nili s.s.* specimens sampled in the deep forest appeared more closely related to *An. ovengensis* than to savannah/degraded forest populations of *An. nili s.s.*

Sequence divergence between consensus haplotypes from each population ranged from 0.4 to 6.2% and from 0.5 to 11.2% for *COII* and *ND4* genes, respectively (Table S3). Because the conventional mtDNA rate of molecular evolution of 2% per myr is used exclusively for lineages separated by less than 8% sequence divergence, only *COII* divergence time was estimated and found to vary approximately between 0.2 and 3 myr. All new mtDNA sequences have been deposited to GenBank (Accession numbers: KC189974 – KC190023).

## Discussion

Genotypes at 11 microsatellite loci and sequence variation in two rDNA regions (D3, ITS2) and two mtDNA genes (*COII*, *ND4*) were used to assess the level of genetic variability and differentiation among species of the *An. nili* group, and between *An. nili s.s.* populations from different ecological settings in South Cameroon. All the markers provided support for the current classification in the group and revealed further cryptic genetic diversity within the major vector *An. nili s.s.*, despite morphological similarities.

Heterologous microsatellite loci are commonly used across many insect species, particularly in *Anopheles* [51]–[54]. In fact, the use of primers originally designed to amplify DNA from a species in a different one can save time and resources, especially for microsatellite loci. However, depending on the genetic distance between species, microsatellite loci could either readily amplify, fail to amplify or show low polymorphism due to the presence of mismatches in priming sites within flanking sequences or absence of nucleotide repeat units variability [52], [55]–[57]. Therefore, it is advocated to screen heterologous loci in few samples to detect suitable markers, before their use in large population genetic studies.

In the present study, 11 *An. nili s.s.* loci were screened for use in *An*. *carnevalei*, *An. somalicus* and *An. ovengensis* and up to 70% cross-amplified in all species. This provides a clear indication that the four species are closely related, since the probability of cross-amplification of microsatellite loci is inversely related with genetic distance between taxa [55], [56]. However, the large differences in microsatellite allele frequencies distribution and sequence profiles, resulting in high pairwise genetic differentiation indices and low migration rates fully support speciation between *An. nili s.s.*, *An*. *carnevalei*, *An. somalicus* and *An. ovengensis*, in agreement with previous studies using allozyme and rDNA markers [8], [58]. Besides, although a high proportion of *An. nili s.s.* loci amplified in the sibling species of the group, only 6 in both *An. carnevalei* and *An. ovengensis*, and 5 in *An. somalicus* showed the qualities that make them suitable genetic markers (e.g., lack of null alleles, high genetic variability, conformity to HWE, statistical independence) and could be used for population genetic studies in these species.

Previously unrecognized cryptic genetic diversity not correlated to geographical distance between sampling sites was evidenced within the nominal species of the group *An. nili s.s.*, by detecting highly divergent lineages in the deep equatorial forest, despite morphological similarities of all specimens analyzed. This genetic substructure was detected by a range of molecular markers with different evolutionary dynamics, providing strong support for phylogenetic inferences. Indeed, rDNA haplotypes found in these populations differed from one another, and from the *An. nili s.s.* haplotypes from savannah/degraded forest areas by a large number of fixed mutations and indels leading to genetic distance estimates 4 to 8 fold higher than those commonly reported among cryptic *Anopheles* species [8], [59]–[64]. According to accepted rates of molecular evolution of 2.2% per myr for ITS2 [48] and 2% per myr for mtDNA [49], we estimated the divergence time of the new molecular forms (lineages) at about 0.8 to 6 Myr or 0.2 to 3 Myr, respectively. This high genetic divergence within the *An. nili* group suggests that these taxa might actually belong to distinct species groups. However, these results generated using a small sample size and on a limited sampling range should be considered as preliminary. Extending the study area throughout the equatorial forest block of Central Africa will allow improving knowledge on the taxonomic status and the geographic distribution of forest *An. nili* s.l. populations, their ecology and behaviour, and their epidemiological role in malaria parasite transmission.

The evergreen forest block of Central Africa is known to be of low overall quality for the development of *An. nili s.s.* which is replaced in this area by the other members of the group, namely *An. carnevalei* and *An. ovengensis* [9], [18], [65]. *Anopheles nili s.s.* is rather more frequent at the savannah/degraded forest ecotone, where it is characterized by shallow population structure with a weak effect of geographical distance on genetic differentiation [18]. Violation of MDE in deep forest populations suggests unstable demographic history for the species in the evergreen forest block of Central Africa. The low dispersal of *An. nili s.s.* in this environment probably restricts opportunities for genetic exchange between geographically isolated populations, prompting for genetic divergence under the action of increased genetic drift [18], [66]. This is in agreement with the low genetic variability observed in forest populations suggesting small effective population size, coupled with high levels of genetic differentiation corresponding to weak migration index estimated between populations. Moreover, the fact that many of the microsatellite markers that were polymorphic in samples from Ako and Nkolbisson did not amplify at all or were not polymorphic (i.e. only one allele could be detected) in deep forest populations is also consistent with the action of increased genetic drift in the later populations. Finally, by demonstrating physical independence between markers, recent mapping of microsatellite loci on the chromosomal arms of *An. nili* provided further support for a major role of genetic drift in shaping the population genetic structure of *An. nili* in Central Africa, reflected in genome-wide differentiation patterns [27]. The development of additional molecular markers is now needed to allow more fine-grained analysis of the level of genetic polymorphism and its distribution along the genome of *An. nili* in search for signatures of selection in ecologically-relevant genes or genomic regions. Indeed, original information gained on the genetic structure, gene flow and species diversity within this major group of malaria vectors can further be used to explore global processes such as the genetics of ecological adaptation, speciation and susceptibility to *Plasmodium*, within a comparative framework that will use and complement information available for other major human malaria vectors such as mosquitoes from the *An. gambiae* complex and the *An. funestus* group.

Moreover, the recent findings of circulation of *P. falciparum* along with other *Plasmodium* species in great apes in Central African forest region [24], [25] raise concerns about pathogen transfer between humans and primates and highlight the need to improve knowledge of forest malaria vectors. Although it is not yet known whether the gorilla carriers of *P. falciparum* acquired the parasite from humans (or from other primates), nor the pathogenicity of these infections in gorillas, the continuously increasing contact between humans and primate populations, mostly due to logging and deforestation, increases opportunities for transmission of new pathogens from primates to humans and vice versa [25]. The possibility that forest mosquitoes such as members of the *An. nili* group might contribute to the emergence of zoonosis in humans needs to be assessed.

## Supporting Information

Figure S1 Alignment of D3 sequences of *An. nili* s.l. haplotypes. AN: *An. nili s.s*.; AO: *An. ovengensis*; AC: *An. carnevalei*; AS: *An. somalicus*. AK: Ako; NK: Nkolbisson; KT: Kentzou; MOA: Moloundou A; MOB: Moloundou B; EK: Ekelemba; AE: Afan-Essokyé; NY: Nyabessan; MB: Mbébé.
(PDF)

Figure S2 **Alignment of ITS2 sequences of** ***An. nili*** **s.l. haplotypes.** AN: *An. nili s.s*.; AO: *An. ovengensis*; AC: *An. carnevalei*; AS: *An. somalicus*. AK: Ako; NK: Nkolbisson; KT: Kentzou; MOA: Moloundou A; MOB: Moloundou B; EK: Ekelemba; AE: Afan-Essokyé; NY: Nyabessan; MB: Mbébé.
(PDF)

Table S1 **Estimates of null alleles frequencies per locus and per geographical population of** ***An. nili*** **s.l. from Cameroon.**
(DOCX)

Table S2 **Pairwise genetic distance estimates between** ***An. nili*** **s.l. D3 (above the diagonal) and ITS2 (below the diagonal) haplotypes from Cameroon.**
(DOCX)

Table S3 **Pairwise genetic distance estimates between** ***An. nili*** **s.l.** ***ND4*** **(above the diagonal) and** ***COII*** **(below the diagonal) haplotypes from Cameroon.**
(DOCX)

## References

[1] World Health Organization (WHO). World malaria report 2011 (2011) Available on http:/www.who.int/malaria/world_malaria_report_2011.

[2] Fontenille D, Simard F (2004) Unravelling complexities in human malaria transmission dynamics in Africa through a comprehensive knowledge of vector populations. Comp Immun Microbiol Infect Dis 27: 357–375. 10.1016/j.cimid.2004.03.005 15225985

[3] Pates H, Curtis C (2005) Mosquito behavior and vector control. Ann Rev Entomol 50: 53–70. 15355233 10.1146/annurev.ento.50.071803.130439

[4] Greenwood B (2009) Can malaria be eliminated? Trans Roy Soc Trop Med Hyg 103: S2–S5. 19062058 10.1016/j.trstmh.2008.10.027

[5] Gillies MT, De Meillon B (1968) The Anophelinae of Africa South of the Sahara (Ethiopian Zoogeographical Region), 2nd ed. The South African Institute for Medical Research, Johannesburg.

[6] Brunhes G, Le Goff G, Geoffroy B (1999) Afro-tropical anopheline mosquitoes. III. Description of three new species: Anopheles carnevalei sp. Nov., and A. hervyi sp. Nov., and A. dualaensis sp. Nov. and resurrection of A. rageaui Mattingly and Adam. J Am Mosq Control Assoc 15 (4): 552–558. 10612618

[7] Awono-Ambene HP, Kengne P, Simard F, Antonio-Nkondjio C, Fontenille D (2004) Description and bionomics of Anopheles (Cellia) ovengensis (Diptera: Culicidae) a new malaria vector species of the Anopheles nili group from south Cameroon. J Med Entomol 41: 561–568. 15311444 10.1603/0022-2585-41.4.561

[8] Kengne P, Awono-Ambene HP, Antonio-Nkondjio C, Simard F, Fontenille D (2003) Molecular identification of the Anopheles nili group African malaria vectors. Med Vet Entomol 17: 67–74. 12680928 10.1046/j.1365-2915.2003.00411.x

[9] Antonio-Nkondjio C, Ndo C, Costantini C, Awono-Ambene P, Fontenille D, Simard F (2009) Distribution and larval habitat characterization of Anopheles nili and An. moucheti along river networks in south Cameroon. Acta Trop 112: 270–276. 19682965 10.1016/j.actatropica.2009.08.009

[10] Hamon J, Mouchet J (1961) Les vecteurs secondaires du paludisme humain en Afrique. Med Trop 21 643–60.

[11] Carnevale P, Le Goff G, Toto JC, Robert V (1992) Anopheles nili as the main vector of human malaria in villages of southern Cameroon. Med Vet Entomol 6: 135–8. 1421483 10.1111/j.1365-2915.1992.tb00590.x

[12] Antonio-Nkondjio,C, Awono-Ambene P, Toto JC, Meunier JY, Zebaze-Kemleu S, et al (2002a) High malaria transmission intensity in a village close to Yaoundé, the capital city of Cameroon. J Med Entomol 39: 350–355. 11931035 10.1603/0022-2585-39.2.350

[13] Dia I, Diop T, Rakotoarivony I, Kengne P, Fontenille D (2003) Bionomics of Anopheles gambiae Giles, A. arabiensis Patton, A. funestus Giles and A. nili (Theobald) (Diptera: Culicidae) and transmission of Plasmodium falciparum in a Sudano-Guinean zone (Ngari, Senegal). J Med Entomol 40: 279–83. 12943105 10.1603/0022-2585-40.3.279

[14] Adja AM, N’goran KE, Kengne P, Koudou GB, Toure M, et al (2006) Transmission vectorielle du paludisme en savane arborée à Gansé en Côte d’Ivoire. Med Trop 66: 449–455. 17201288

[15] Adja AM, N’goran EK, Koudou GB, Dia I, Kengne P, et al (2011) Contribution of Anopheles funestus, An. gambiae and An. nili (Diptera: Culicidae) to the perennial malaria transmission in the southern and western forest areas of Cote d’Ivoire. Ann Trop Med Parasitol 105(1): 13–24. 21294945 10.1179/136485910X12851868780388 PMC4089788

[16] Awono-Ambene P, Antonio-Nkondjio C, Toto JC, Ndo C, Etang J, et al (2009) Epidemological importance of the Anopheles nili group of malaria vectors in equatorial villages of Cameroon, Central Africa. Sci Med Afr 1: 13–20.

[17] Ridl FC, Bass C, Torrez M, Govender D, Ramdeen V, et al (2008) A pre-intervention study of malaria vector abundance in Rio Muni, Equatorial Guinea: their role in malaria transmission and the incidence of insecticide resistance alleles. Malar J 7: 194. 18823554 10.1186/1475-2875-7-194 PMC2564967

[18] Ndo C, Antonio-Nkondjio C, Cohuet A, Ayala D, Kengne P, et al (2010) Population genetic structure of the malaria vector Anopheles nili in sub-Saharan Africa. Malar J 9: 161. 20540796 10.1186/1475-2875-9-161 PMC2898787

[19] Rivola E, Holstein MH (1957) Note sur une variété d'Anopheles nili Theo. Bull Soc Path Exo 50: 382–387. 13479752

[20] Antonio-Nkondjio C, Kerah Hinzoumbe C, Simard F, Awono-Ambene P, Tchuinkam T, et al (2006) Complexity of the malaria vectorial system in Cameroon: Contribution of secondary vectors to malaria transmission. J Med Entomol 43: 1215–1221. 17162956 10.1603/0022-2585(2006)43[1215:cotmvs]2.0.co;2

[21] Ollomo B, Durand P, Prugnolle F, Douzery E, Arnathau C, et al (2009) A new malaria agent in African hominids. PLoS Pathog 5: e1000446. 19478877 10.1371/journal.ppat.1000446 PMC2680981

[22] Duval L, Fourment M, Nerrienet E, Rousset D, Sadeuh SA, et al (2010) African apes as reservoirs of Plasmodium falciparum and the origin and diversification of the Laverania subgenus. Proc Natl Acad Sci USA 107: 10561–10566. 20498054 10.1073/pnas.1005435107 PMC2890828

[23] Liu W, Li Y, Learn GH, Rudicell RS, Robertson JD, et al (2010) Origin of the human malaria parasite Plasmodium falciparum in gorillas. Nature 467: 420–5. 20864995 10.1038/nature09442 PMC2997044

[24] PrugnolleF, Durand P, Neel C, Ollomo B, Ayala FJ, et al (2010) African great apes are natural hosts of multiple related malaria species, including Plasmodium falciparum. Proc Natl Acad Sci USA 107: 1458–63. 20133889 10.1073/pnas.0914440107 PMC2824423

[25] Prugnolle F, Ollomo B, Durand P, Yalcindag E, Arnathau C, et al (2011) African monkeys are infected by Plasmodium falciparum nonhuman primate-specific strains. PNAS 108 (29): 11948–11953. 10.1073/pnas.1109368108 PMC3141972 21730135

[26] Sharakhova MV, Antonio-Nkondjio C, Xia A, Ndo C, Awono-Ambene P, et al (2011) Cytogenetic map for Anopheles nili: Application for population genetics and comparative physical mapping. Infect Genet Evol 11: 746–754. 20603229 10.1016/j.meegid.2010.06.015 PMC3036789

[27] Peery A, Sharakhova M, Antonio-Nkondjio C, Ndo C, Weill M, et al (2011) Improving the population genetics toolbox for the study of the African malaria vector Anopheles nili: microsatellite mapping chromosomes. Para Vect 4: 202. 10.1186/1756-3305-4-202 PMC3222614 22011455

[28] Gillies MT, Coetzee M (1987) A Supplement to the Anophelinae of Africa south of the Sahara. South African Institute of Medical Research, Johannesburg, p. 143.

[29] Morlais I, Ponçon N, Simard F, Cohuet A, Fontenille D (2004) Intraspecific nucleotide variation in Anopheles gambiae: new insights into the biology of malaria vectors. Am J Trop Med Hyg 71(6): 795–802. 15642974

[30] Berthomieu A, Kengne P, Awono-Ambene HP, Raymond M, Fontenille D, et al (2003) Isolation and characterization of microsatellite DNA markers in the malaria vector Anopheles nili. Mol Ecol Notes 3: 392–393.

[31] El Mousadik A, Petit R (1996) High level of genetic differentiation for allelic richness among populations of the argan tree (Argania spinosa L.) Skeels endemic to Morocco. Theo Appl Gen (92): 832–839. 10.1007/BF00221895 24166548

[32] Goudet J (1995) FSTAT version 2.9.3.2. A computer software to calculate F-statistics. J Hered 86: 485–486.

[33] Raymond M, Rousset F (1995) GENEPOP, Version 1.2. A population genetics software for exact tests and ecumenicism. J Hered 86: 248–249.

[34] Rousset F (2007) Genepop’007 a complete re-implementation of the Genepop software for windows and linux. Mol Ecol Notes 8: 103–106. 10.1111/j.1471-8286.2007.01931.x 21585727

[35] Van Oosterhout C, Hutchinson WF, Wills DPM, Shipley P (2004) MICROCHECKER: Software for identifying and correcting genotyping errors in microsatellite data. Mol Ecol 4: 535–538.

[36] Cornuet JM, Luikart G (1996) Description and power analysis of two tests for detecting recent population bottlenecks from allele frequency data. Genetics 144: 2001–2014. 8978083 10.1093/genetics/144.4.2001 PMC1207747

[37] Pritchard JK, Stephens M, Donnelly P (2000) Inference of population structure using multilocus genotype data. Genetics 155: 945–959. 10835412 10.1093/genetics/155.2.945 PMC1461096

[38] Evanno G, Regnaut S, Goudet J (2005) Detecting the number of clusters of individuals using the software STRUCTURE: a simulation study. Mol Ecol 14: 2611–2620. 15969739 10.1111/j.1365-294X.2005.02553.x

[39] Wright S (1978) Evolution and Genetics of populations. Variability within and among natural populations. Vol 4, Chicago, University of Chicago press.

[40] Weir BS, Cockerham CC (1984) Estimating F-statistics for the analysis of population structure. Evolution 38: 1358–1370. 28563791 10.1111/j.1558-5646.1984.tb05657.x

[41] Slatkin M (1995) A measure of population subdivision based on microsatellite allele frequencies. Genetics 139: 457–462. 7705646 10.1093/genetics/139.1.457 PMC1206343

[42] Rousset F (1997) Genetic differentiation and estimation of gene flow from F-statistics under isolation by distance. Genetics 145(4): 1219–1228. 9093870 10.1093/genetics/145.4.1219 PMC1207888

[43] Rousset F (2007) Genepop’007 a complete re-implementation of the Genepop software for windows and linux. Mol Ecol Notes 8: 103–106. 10.1111/j.1471-8286.2007.01931.x 21585727

[44] Holm S (1979) A simple sequentially rejective multiple test procedure. Scand J Statt 6: 65–70.

[45] Librado P, Rozas J (2009) DnaSP v5: a software for comprehensive analysis of DNA data. Bioinformatics 25: 1451–1452. 19346325 10.1093/bioinformatics/btp187

[46] Kumar S, Tamura K, Nei M (2004) MEGA 3: integrate software for Molecular Evolutionary Genetics Analysis and sequence alignment. Brief Bioinformatics 5: 150–163. 15260895 10.1093/bib/5.2.150

[47] Tajima F, Nei M (1984) Estimation of evolutionary distance between nucleotide sequences. Mol Biol Evol1 (3): 269–85. 10.1093/oxfordjournals.molbev.a040317 6599968

[48] Schlötterer C, Hausser MT, Von Haeseler A, Tautz D (1994) Comparative evolutionary analysis of rDNA ITS regions in Drosophila. Mol Biol Evol 11 (3): 513–522. 10.1093/oxfordjournals.molbev.a040131 8015444

[49] Desalle R, Templeton A, Mori I, Pletscher S, Johnston JS (1987) Temporal and spatial heterogeneity of mtDNA polymorphism in natural populations of Drosophila mercatorum. Genetics 116: 215–223. 3038671 10.1093/genetics/116.2.215 PMC1203132

[50] Saitou N, Nei M (1987) The neighbor-joining method: a new method for reconstructing phylogenetic trees. Mol Biol Evol 4: 406–425. 3447015 10.1093/oxfordjournals.molbev.a040454

[51] Walton C, Thelwell TJ, Priestman A, Butlin RK (1998) The use of microsatellites to study gene flow in natural populations of Anopheles malaria vectors in Africa: potential and pitfalls. J Am Mosq. Control Assoc 14: 266–272. 9813823

[52] Kamau L, Lehmann T, Hawley WA, Orago ASS, Ke Z, et al (1998) Use of short tandem repeats for analysis of genetic variability in sympatric populations of An. gambiae and An. arabiensis. Heredity 80: 675–682. 9675871 10.1046/j.1365-2540.1998.00327.x

[53] Simard F, Fontenille D, Lehmann T, Girod R, Brutus L (1999) High amounts of genetic differentiation between populations of the malaria vector Anopheles arabiensis from West Africa and eastern outer islands. Am J Trop Med Hyg 60: 1000–1009. 10403334 10.4269/ajtmh.1999.60.1000

[54] Antonio-Nkondjio C, Ndo C, Kengne P, Mukwaya L, Awono-Ambene HP, et al (2008) Population structure of the malaria vector Anopheles moucheti in the equatorial forest region of Africa. Malar J 7: 120. 18601716 10.1186/1475-2875-7-120 PMC2483286

[55] Weber JL, Kwitek AE, May PE, Killary AM (1990) Dinucleotide repeat polymorphism at the, D4s 174-locus. Nucl Acids Res 18: 4636–4638. PMC331327 2388858

[56] Primmer CR, Møller AP, Ellegren H (1996) A wide-range survey of cross-species microsatellite amplification in birds. Mol Ecol 5: 365–378. 8688957 10.1111/j.1365-294x.1996.tb00327.x

[57] Deitz KC, Reddy VP, Reddy MR, Satyanarayanah N, Lindsey, et al (2012) Limited usefulness of microsatellite markers from the malaria vector Anopheles gambiae when applied to the closely related species Anopheles melas. J Hered 103 (4): 585–593. 10.1093/jhered/ess025 22593601

[58] Awono-Ambene HP, Simard F, Antonio-Nkondjio C, Cohuet A, Kengne P, et al (2006) Multilocus enzyme electrophoresis supports speciation within the Anopheles nili group of Malaria vectors in Cameroon. Am J Trop Med Hyg 75: 656–658. 17038689

[59] Paskewitz SM, Ng K, Coetzee M, Hunt RH (1993) Evaluation of the polymerase chain reaction method for identifying members of the Anopheles gambiae (Diptera: Culicidae) complex in Southern Africa. J Med Entomol 30: 953–957. 8254648 10.1093/jmedent/30.5.953

[60] Scott JA, Brogdon WG, Collins FH (1993) Identification of single specimens of the Anopheles gambaie complex by the polymerase chain reaction. Am J Trop Med Hyg 49 (4): 520–529. 10.4269/ajtmh.1993.49.520 8214283

[61] Collins FH, Paskewitz SM (1996) A review of the use of ribosomal DNA (rDNA) to differentiate among cryptic Anopheles species. Ins Mol Biol 5 (1): 1–9. 10.1111/j.1365-2583.1996.tb00034.x 8630529

[62] Cohuet A, Simard F, Toto JC, Kengne P, Coetzee M, et al (2003) Species identification within the Anopheles funestus group of malaria vectors in Cameroon and evidence for a new species. Am J Trop Med Hyg 69 (2): 200–205. 13677376

[63] Kengne P, Antonio-Nkondjio C, Awono-Ambene HP, Simard F, Awolola TS, et al (2007) Molecular differentiation of three closely related members of the mosquito species complex, Anopheles moucheti, by mitochondrial and ribosomal DNA polymorphism. Med Vet Entomol 21: 177–182. 17550437 10.1111/j.1365-2915.2007.00681.x

[64] Manonmani AM, Sadanandane C, Sahu SS, Mathivanan A, Jambulingam P (2007) rDNA-ITS2-PCR assay for groupping the cryptic species of Anopheles culicifacies complex (Diptera: Culicidae). Acta Trop 104 (1): 72–77. 10.1016/j.actatropica.2007.07.002 17709089

[65] Ayala D, Costantini C, Ose K, Kamdem GC, Antonio-Nkondjio C, et al (2009) Habitat suitability and ecological niche profile of major malaria vectors in Cameroon. Malar J 8: 307. 20028559 10.1186/1475-2875-8-307 PMC2805691

[66] Le Goff G, Carnevale P, Robert V (1997) Low dispersion of anopheline malaria vectors in the african equatorial forest. Parasite 2: 187–189.

[67] Silver JB (2008) Mosquito ecology: field sampling methods. Springer, 3rd Edition.

