# Supplementary material for: Cryptic Genetic Diversity within the Anopheles nili group of Malaria Vectors in the Equatorial Forest Area of Cameroon (Central Africa)
Source: PLoS One. 2013 Mar 14;8(3):e58862. doi: 10.1371/journal.pone.0058862 (PMC3597579; doi:10.1371/journal.pone.0058862)
Supplement: Figure S1 — Alignment of D3 sequences of An. nili s.l. haplotypes. AN: An. nili s.s.; AO: An. ovengensis; AC: An. carnevalei; AS: An. somalicus. AK: Ako; NK: Nkolbisson; KT: Kentzou; MOA: Moloundou A; MOB: Moloundou B; EK: Ekelemba; AE: Afan-Essokyé; NY: Nyabessan; MB: Mbébé. (PDF) [file pone.0058862.s001.pdf]

```
                            111 111 111 122 222 222 223 333 333 333 444 444 444 455 555 555 556 666 666 666 777 777 777
                123 456 789 012 345 678 901 234 567 890 123 456 789 012 345 678 901 234 567 890 123 456 789 012 345 678
AN (AK)         CTA CTA GAT GGT TCG ATT GGT CTT TCG CCC CTA TGC CCA ATT CTG ACA ATC GAT TTG CAC GTC AGA ATT GCT TCG GTC
AN (NK)         ... ... ... ... ... ... ... ... ... ... ... ... ... ... ... ... ... ... ... ... ... ... ... ... ... ...
AN (KT)         ... ... ... ... ... ... ... ... ... ... ... ... ... ... ... ... ... ... ... ... ... ... ... ... ... ...
AN (MOA)        ... ... ... ... ... ... ... ... ... ... ... ... ... ... ... ... ... ... ... ... ... ... ... ... ... ...
AN (MOB)        ... ... ... ... ... ... ... ... ... ... ... ... ... ... ... ... ... ... ... ... ... ... ... ... ... ...
AN (EK)         ... ... ... ... ... ... ... ... ... ... ... ... ... ... ... ... ... ... ... ... ... ... ... ... ... ...
AC (AE)         ... ... ... ... ... ... ... ... ... ... ... ... ... ... ... ... ... ... ... ... ... ... ... ... ... ...
AO (NY)         ... ... ... ... ... ... ... ... ... ... ... ... ... ... ... ... ... ... ... ... ... ... ... ... ... ...
AS (MB)         ... ... ... ... ... ... ... ... ... ... ... ... ... ... ... ... ... ... ... ... ... ... ... ... ... ...

                                            111 111 111 111 111 111 111 111 111 111 111 111 111 111 111 111 111 111 111
                788 888 888 889 999 999 999 000 000 000 011 111 111 112 222 222 222 333 333 333 344 444 444 445 555 555
                901 234 567 890 123 456 789 012 345 678 901 234 567 890 123 456 789 012 345 678 901 234 567 890 123 456
AN (AK)         CTC CAT CAG GGT TTC CCC TGA CTT CAA CCT GAT CAG GCA TAG TTC ACC ATC TTT CGG GTC GCA TCC TAC GCA CTC GGG
AN (NK)         ... ... ... ... ... ... ... ... ... ... ... ... ... ... ... ... ... ... ... ... ... ... ... ... ... ...
AN (KT)         ... ... ... ... ... ... ... ... ... ... ... ... ... ... ... ... ... ... ... ... ... ... ... ... ... ...
AN (MOA)        ... ... ... ... ... ... ... ... ... ... ... ... ... ... ... ... ... ... ... ... ... ... ... ... ... ...
AN (MOB)         ... ... ... ... ... ... ... ... ... ... ... ... ... ... ... ... ... ... ... ... ... ... ... ... ... ...
AN (EK)         ... ... ... ... ... ... ... ... ... ... ... ... ... ... ... ... ... ... ... ... ... ... ... ... ... ...
AC (AE)         ... ... ... ... ... ... ... ... ... ... ... ... ... ... ... ... ... ... ... ... ... ... ... ... ... ...
AO (NY)         ... ... ... ... ... ... ... ... ... ... ... ... ... ... ... ... ... ... ... ... ... ... ... ... ... ...
AS (MB)         ... ... ... ... ... ... ... ... ... ... ... ... ... ... ... ... ... ... ... ... ... ... ... ... ... ...

                111 111 111 111 111 111 111 111 111 111 111 111 111 111 122 222 222 222 222 222 222 222 222 222 222 222
                555 666 666 666 677 777 777 778 888 888 888 999 999 999 900 000 000 001 111 111 111 222 222 222 233 333
                789 012 345 678 901 234 567 890 123 456 789 012 345 678 901 234 567 890 123 456 789 012 345 678 901 234
AN (AK)         GGA TGC CCG CTG GGT GGC GCA CGT GTG ACC GCA CGC CAG CCC GTA CCG GGG CAC CCT GGG ATG GAG GGA GGC GTC CGT
AN (NK)         ... ... ... ... ... ... ... ... ... ... ... ... ... ... ... ... ... ... ... ... ... ... ... ... ... ...
AN (KT)         ... ... ... ... ... ... ... ... ... ... ... ... ... ... ... ... ... ... ... ... ... ... ... ... ... ...
AN (MOA)        ... ... ... ... ... ... ... ... ... ... ... ... ... ... ... ... ... ... ... ... ... ... ... ... ... ...
AN (MOB)        ... ... ... ... ..C ... ... ... ... ... ... ... ... ... ..G A.. ... ... ... ... ... ... ... ... ... .A.
AN (EK)         ... ... ... ... ..C ... ... ... T.. ... ... ... ... ... ..G A.. ... ... ... ... ... ... ... ... ... .A.
AC (AE)         ... ... ... ... ... ... ... ... ... ... ... ... ... ... ... ... ... ... ... ... ... ... ... ... ... ...
AO (NY)         ... ... ... ... ... ... ... ... ... ... ... ... ... ... ... ... ... ... ... ... ... ... ... ... ... ...
AS (MB)         ... ... ... ... ... ... ... ... ... ... ... ... ... ... ... ... ... ... ... ... ... ... ... ... ... ...
```

Figure S1.

```
             222 222 222 222 222 222 222 222 222 222 222 222 222 222 222 222 222 222 222 222 222 223 333 333 333 333
             333 334 444 444 444 555 555 555 566 666 666 667 777 777 777 888 888 888 899 999 999 990 000 000 000 111
             567 890 123 456 789 012 345 678 901 234 567 890 123 456 789 012 345 678 901 234 567 890 123 456 789 012
AN (AK)      GGC TTG CGC CAA GCG CGC CCC CGT AAT CCC GCG ACG AAA CCG TCT CGA GTT GTC TGC GCC TGT GGG GTT CTC TCT CGC
AN (NK)      ... ... ... ... ... ... ... ... ... ... ... ... ... ... ... ... ... ... ... ... ... ... ... ... ... ...
AN (KT)      ... ... ... ... ... ... ... ... ... ... ... ... ... ... ... ... ... ... ... ... ... ... ... ... GG- TAT
AN (MOA)     ... ... ... ... ... ... ... ... ... ... ... ... ... ... ... ... ... ... ... ... ... ... ... ... GG- TAT
AN (MOB)     ... ... .A. ... ... ... ... ... ... ... ... ... ... ... ... ... ... ... ... ... ... ... ... ... GG- T.T
AN (EK)      ... ... .A. ... ... ... ... ... ... ... ... ... ... ... ... ... ... ... ... ... ... ... ... ... GG- T.T
AC (AE)      ... ... ... ... ... ... ... ... ... ... ... ... ... ... ... ... ... ... ... ... ... ... ... ... GG. T.-
AO (NY)      ... ... ... ... ... ... ... ... ... ... ... ... ... ... ... ... ... ... ... ... ... ... ... ... GG- T..
AS (MB)      ... ... ... ... ... ... ... ... ... ... ... ... ... ... ... ... ... ... ... ... ... ... ... ... GTC T.T

             333 333 333 333 333 333 333 333 333 333 333 333 333 333 333 333 333 333 333 333 333 333 333 3
             111 111 122 222 222 223 333 333 333 444 444 444 455 555 555 556 666 666 666 777 777 777 788 8
             345 678 901 234 567 890 123 456 789 012 345 678 901 234 567 890 123 456 789 012 345 678 901 2
AN (AK)      GGT ATA CTG GGG C-- --- GCA AGC --G CCC CAA CAA CCT GGC CCA TTG GCT CGC GCG TAA GAT AGA CTT C
AN (NK)      ... ... ... ... .-- --- ... ... --. ... ... ... ... ... ... ... ... ... ... ... ... ... ... .
AN (KT)      AC. GGG GC. ..C .CA CAC .G. T.. GG. T.. GCG .CC .AA C.. ... ... ... ... ... ... ... ... ... .
AN (MOA)     AC. GGG GC. ..C TTG --- --- --- --- -.. GCG .CC .AA C.. ... ... ... ... ... ... ... ... ... .
AN (MOB)     AC. GGG G.. C.C --- --- ..G ... ACA ... ... .CG .-- --- ... ... ... ... ... ... ... ... ... .
AN (EK)      AC. TGG G.. C.C --- --- ..G T.. GC. ... ... .CG .GC --- ... ... ... ... ... ... ... ... ... .
AC (AE)      -C. GGG TC. .AC .CA C-- .G. T.- -G. T.. GC- .C. .GC C.. ... ... ... ... ... ... ... ... ... .
AO (NY)      AC. GGG GC. ..C .CA CAC .G. T.. GG. T.. GCG .CC .AA C.. ... ... ... ... ... ... ... ... ... .
AS (MB)      .C. GGG TC. .AC .CG --- .GC ..- -G. T.. GC- .C. .GC C-- -.. ... ... ... ... ... ... ... ... .
```

Figure S1 (continued).
