# Supplementary material for: Cryptic Genetic Diversity within the Anopheles nili group of Malaria Vectors in the Equatorial Forest Area of Cameroon (Central Africa)
Source: PLoS One. 2013 Mar 14;8(3):e58862. doi: 10.1371/journal.pone.0058862 (PMC3597579; doi:10.1371/journal.pone.0058862)
Supplement: Figure S2 — Alignment of ITS2 sequences of An. nili s.l. haplotypes. AN: An. nili s.s.; AO: An. ovengensis; AC: An. carnevalei; AS: An. somalicus. AK: Ako; NK: Nkolbisson; KT: Kentzou; MOA: Moloundou A; MOB: Moloundou B; EK: Ekelemba; AE: Afan-Essokyé; NY: Nyabessan; MB: Mbébé. (PDF) [file pone.0058862.s002.pdf]

```
                        111 111 111 122 222 222 223 333 333 333 444 444 444 455 555 555 556 666 666 666 777 777 777
            123 456 789 012 345 678 901 234 567 890 123 456 789 012 345 678 901 234 567 890 123 456 789 012 345 678
AN (AK)     --- --- --- --- --- --- --- --T CAC ACA TCA CTT GAG GCC TAC --- --- --- --- --C --- --- --- --- --- ---
AN (NK)     --- --- --- --- --- --- --- --. ... ... ... ... ... ... ... --- --- --- --- --. --- --- --- --- --- ---
AN (KT)     --- --- --- --- --- --- --- GC. ... ... ... ... ... ... ... --- --- --- --- --T G-- --- --- --- --- ---
AN (MOA)    --- --- --- --- --- --- --- GC. ... ... ... ... ... ... ... --- --- --- --- --T T-- --- --- --- --- ---
AN (MOB)    --- --- --- --- --- --- --- GC. ... ... ... ... ... ... ... --- --- --- --- --T GTA TGT AAC GTG CCA TGC
AN (EK)     --- --- --- --- --- --- --- GC. ... ... ... ... ... ... ... --- --- --- --- --T GTA TGT AAC GCG TTC CAT
AC (AE)     --- --- --- --- --- --- --- --. ... ... ... ... ... ... ... --- --- --- --- --T G-- --- --- --- --- ---
AO (NY)     --- --- --- --- --- --- --- --. ... ... ... ... ... ... ... A-- --- -TG TAG GTT TCC AAT ATT TAT ATA TAT
AS (MB)     --- --- --- --- --- --- --- --. ... ... ... ... ... ... ... TGT ATA TTA TGT GTT TGT GTT GGT GTT CAC GAC

                                    111 111 111 111 111 111 111 111 111 111 111 111 111 111 111 111 111 111 111
            788 888 888 889 999 999 999 000 000 000 011 111 111 112 222 222 222 333 333 333 344 444 444 445 555 555
            901 234 567 890 123 456 789 012 345 678 901 234 567 890 123 456 789 012 345 678 901 234 567 890 123 456
AN (AK)     -TA AAG GT- --- TAA CTT CAT ATA TGC ACG -CA CAC ACG --- ACG -AG ACG A-- --- --- CGA CCA CGG TCT TGC -C-
AN (NK)     -.. ... ..- --- ... ... ... ... ... ... -.. ... ... --- ... -.. ... .-- --- --- ... ... ... ... ... -.-
AN (KT)     --G T.. ..- --- .TT TCC A.C ..G ..T --- -.G T.. ... --- CTA --- -.T C-- --- --T .-T ... ... ... ... -.-
AN (MOA)    --G T.. ..- --- .TT TCC A.C ..G .-- --- -.G T.. ... --- CTC --- -.T C-- --- --T .-T ... ... ... ... -.-
AN (MOB)    AC. C.C ..- --- CTC TC. T.C ..G C.T ... CAG ... ... --- C.A CTT .TA C-- --- --T ..T ... ... ... ... -.-
AN (EK)     A.G C.C ..- --- CTC TC. T.C ..G C.T ... CAG ... ... --- C.. -TT .TA C-- --- --T .TT ... ... ... ... -.C
AC (AE)     G.G GG. T.- --- CTT ..C TTC TCG .A. ... -A. AG. ..A --- G.A -CC ... CGC T-- --C TCT ... ... ... ... -.T
AO (NY)     A.. T.T A.- --- AT. TA. T.. ... .AT .TC AT. A.T .TA CAC G.. TGC ... CAG CAC GGT .AC .T. ... .TC .C. A.C
AS (MB)     AA. C.C T.G GCG .TT .GC AGC .CG .T. ..C A.. ... CA. CTG CT. CT. CT. GCT GGC TGC TCT .G. T.A G.A CA. GGT

            111 111 111 111 111 111 111 111 111 111 111 111 111 111 122 222 222 222 222 222 222 222 222 222 222 222
            555 666 666 666 677 777 777 778 888 888 888 999 999 999 900 000 000 001 111 111 111 222 222 222 233 333
            789 012 345 678 901 234 567 890 123 456 789 012 345 678 901 234 567 890 123 456 789 012 345 678 901 234
AN (AK)     -AA CCG GC- -GG C-- --- --G -GT GTG TAT GGG CAG C-G CGC GCA -TC GGC ATT GCG TCG TTC GCA CGC GCG CGG TGT
AN (NK)     -.. ... ..- -.. .-- --- --. -.. ... ... ... ... .-. ... ... -.. ... ... ... ... ... ... ... ... ... ...
AN (KT)     -.C .A. .G- --- --- --- --- C.. ... ... ... ... .-. .A. C.. -A. ... ... ... ... .C. ... ... ... ... ...
AN (MOA)    -.C .A. .G- --- --- --- --- C.. ... ... ... ... .-. .A. C.. -A. ... ... ... ... ... ... ... ... ... ...
AN (MOB)    -.C .A. .GA G.. .-- --- --. C.A ... ... ... ... .-. .A. C-. -A. ... ... ... ... ... ... ... ... ... ...
AN (EK)     -.C .A. .GA G.. .-- --- --. C.A ... ... ... ... .-. GA. C-. -A. ... ... ... ... ... ... ... ... ... ...
AC (AE)     C.C .A. .G- -T. .-- --- --. CA. .C. ... ... ... .-A ..- -.. -A. ..T ..A ... ... ... ... .A. ... ..T C..
AO (NY)     T.C A.. .TC TT. .CT CCA GG. C.. ... ... ... ... .-. .A. CA. -A. ... ... ... ... C.. ... ... ... ... ...
AS (MB)     C.T G.A C.A G.. GGT TT- GG. CC. ... ... ..A ... .-. .TT CA. CA. ... ..A ... ... ... ... ... ... ..T C..
```

Figure S2.

```
                222 222 222 222 222 222 222 222 222 222 222 222 222 222 222 222 222 222 222 222 222 223 333 333 333 333
                333 334 444 444 444 555 555 555 566 666 666 667 777 777 777 888 888 888 899 999 999 990 000 000 000 111
                567 890 123 456 789 012 345 678 901 234 567 890 123 456 789 012 345 678 901 234 567 890 123 456 789 012
AN (AK)         AGC --T CCT AGG G-T TAG GTT ACA CGC GGC GTG CC- --T CGG CGA ACC GTG GCG C-T GCT TGA CAC AGC CCC CTG GCT
AN (NK)         ... --. ... ... .-. ... ... ... ... ... ... ..- --. ... ... ... ... ... .-. ... ... ... ... ... ... ...
AN (KT)         ... --. ... ... .-. ... ... ... ... ... ... ..- --. ... ... ... ... ... .-. ... ... ... ... .T. ... ..C
AN (MOA)        ... --. ... ... .-. ... ... ... ... ... ... ..- --. ... ... ... ... ... .-. ... ... ... ... .T. ... ..C
AN (MOB)        .T. --. .G. G.. .G. ... ... ... ... ... ... ..- --. ... ... ... ... ... .-. ... ... ... ... .T. ... ..C
AN (EK)         .T. --. .G. G.. .G. ... ... ... ... ... ... ..- --. ... ... ... ... ... .-. ... ... ... ... .T. ... ..C
AC (AE)         ..T --A ... ... .-. ... .C. ... ... ... ... ..- --C ..T ... ... ... ... .C. ... ... .G. ... ... -.. ..C
AO (NY)         ... --. ... ... .-A ... .AC ... ... ... ... ..- --. ... .A. ... ... ... .-. ... ... ... ... .T. ... ..C
AS (MB)         ..T --A ... ... .-. ... .C. .G. ... ... ... ..- --. ... ... ... ... ... .-. ... ... ... ... ... -.. ...

                333 333 333 333 333 333 333 333 333 333 333 333 333 333 333 333 333 333 333 333 333 333 333 333 333 333
                111 111 122 222 222 223 333 333 333 444 444 444 455 555 555 556 666 666 666 777 777 777 788 888 888 889
                345 678 901 234 567 890 123 456 789 012 345 678 901 234 567 890 123 456 789 012 345 678 901 234 567 890
AN (AK)         GCT TCT CGT GGC GCG GTG CGC GTG CCT --- GCT CCT TCG CAT GTT ATG CTC TCT TCA GC- GCC CCG GGG TGG T-- -AA
AN (NK)         ... ... ... ... ... ... ... ... ... --- ... ... ... ... ... ... ... ... ... ..- ... ... ... ... .-- -..
AN (KT)         ... ... ... ... .-- --. ... ... ..C --- .A. ... ... ... ... C.. --. ... ... ..- ... ... ... ... .-- ---
AN (MOA)        ... ... ... ... .-- --. ... ... ..C --- .A. ... ... ... ... C.. --. .T. ... ..- ... ... ... ... .-- ---
AN (MOB)        ... ... ... ... .-- --. ... ... ..C --- .A. ... ... ... ..G T.. --. ... ... ..- ... ... ... ... .-- ---
AN (EK)         ... ... ... ... .-- --. ... ... ..C --- .A. ... ... ... ..G T.. --. ... ... ..- ... ... ... ... .-- ---
AC (AE)         ... ... ... ... .-- --. ... ... ..C A-- .T. ... ... ... ... G-- --. ... ... ..- ... ... ... ... .GG T..
AO (NY)         ... ... ... ... .-- --. ... .C. ..C G-- ATC .T. CG. ... ... GC. --. ... ... ..A ... ... ... ... .-- ---
AS (MB)         ... ... ... ... .GT .C. .A. ... ..C GAT AAC .T. CTT .GC A.. G.T G-. ... ... ..G C.. .G. ... ... A-- ---

                333 333 333 444 444 444 444 444 444 444 444 444 444 444 444 444 444 444 444 444 444 444 444 444 444 444
                999 999 999 000 000 000 011 111 111 112 222 222 222 333 333 333 344 444 444 445 555 555 555 666 666 666
                123 456 789 012 345 678 901 234 567 890 123 456 789 012 345 678 901 234 567 890 123 456 789 012 345 678
AN (AK)         GTG --- --- --- -AC CG- CCC CAG CAC GCC ATG CTG C-G CTC GTG --- -TG CTG TCA CAC AA- --- -CA ACA CGA GCA
AN (NK)         ... --- --- --- -.. ..- ... ... ... ... ... ... .-. ... ... --- -.. ... ... ... ..- --- -.. ... ... ...
AN (KT)         ... --- --- --- -.. ..A ... ... ... ... ... ... .-. ... ... GCA C.C .C. .GT GTT GGG CGC A.C .GC -.. ...
AN (MOA)        ... --- --- --- -.. ..A ... ... ... ... ... ... .-. ... ... GCA C.C .C. .GT GTT GGG CGT A.C .GC -.. ...
AN (MOB)        ... --- --- --- -.. ..- ... ... ... ... ... ... .-. ... ... GCA C.C .C. .GT GT. GGG CGT A.C GGC ... ...
AN (EK)         ... --- --- --- -.. ..- ... ... ... ... ... ... .-. ... ... GCG CAC .C. .GT GT. GGG CGT A.C GGC ... ...
AC (AE)         ... TTG TAA CCT G.. .C- ... ... ... ... ... ... .T. ... ... GTG A.. A.. C.. AG. CGG CC- -.C ..C ... ...
AO (NY)         ... --- --- --- -.. ..- ... .G. ... ... ... ... .-. ... ... GCA CCC .C. .GT GGT GGG CGT A.C .GC -.. ...
AS (MB)         C.. --- --- --- -.. ..- ... ... ... ... ... ... .-. ... ... --- --- --. CGT AG. CG- --- -.C .TC -.. ...
```

Figure S2 (continued).

```
                 444 444 444 444 444 444 444 444 444 444 455 555 555 555 555 555 555 555 555 555 555 555 555 555 555 555
                 677 777 777 778 888 888 888 999 999 999 900 000 000 001 111 111 111 222 222 222 233 333 333 334 444 444
                 901 234 567 890 123 456 789 012 345 678 901 234 567 890 123 456 789 012 345 678 901 234 567 890 123 456
AN (AK)          GTC TGA GC- --- --C AAC GCT T-- GTC TCA ACA ATT GAG TAG GCA CTC AAG AAT GTG TGC ATC GGG CGG GTT GAA GCG
AN (NK)          ... ... ..- --- --. ... ... .-- ... ... ... ... ... ... ... ... ... ... ... ... ... ... ... ... ... ...
AN (KT)          ... ... ..C --- -AT --A ... .-- .C. ... ... ..C ... ... ... ... ... ... ... ... ... ... ... ... ... ...
AN (MOA)         ... ... .TC --- -AT .TA ... .-- ... ... ... ..C ... ... ... ... ... ... ... ... ... ... ... ... ... ...
AN (MOB)         ... ... ..C --- -AT .-G ... .-- ... ... ... ..C ... ... ... ... ... ... ... ... ... ..T ... ... ... A..
AN (EK)          ... ... ..C --- -AT T-G ... .-- ... ... ... ..C ... ... ... ... ... ... ... ... ... ... ... ... ... ...
AC (AE)          ... ... .TG TGT AGT .G. AT. .TT ... ... ... ..C ... ... ... ... ... ... ... ... ... ... ... ... ... ...
AO (NY)          ... ... ..- --- --. .TA ... .-- ... .-. ... ..C ... ... ... ... ... ... ... ... ... ... ... ... ... ...
AS (MB)          ... ... ..G CGC AAT G-A ... .-- ... ... ... ..C ... ... ... ... ... ... ... ... ... ... ... ... ... ...

                 555 555 555 555 555 555 555 555 555 555 555 555 5
                 444 555 555 555 566 666 666 667 777 777 777 888 8
                 789 012 345 678 901 234 567 890 123 456 789 012 3
AN (AK)          TCC GAT GCG CCA TAT GCG TT- CAA CGT GTC GGT GTT C
AN (NK)          ... ... ... ... ... ... ..- ... ... ... ... ... .
AN (KT)          ... ... ... ... ... ... ..- ... ... ... ... ... .
AN (MOA)         ... ... ... ... ... ... ..- ... ... ... ... ... .
AN (MOB)         ... ... ... ... ... ... ..- ... ... ... ... ... .
AN (EK)          ... ... ... ... ... ... ..- ... ... ... ... ... .
AC (AE)          ... ... ... ... ... ... ..- ... ... ... ... ... .
AO (NY)          -.. ... ..A ... ... ... .-- ... ... ... ... ... .
AS (MB)          ... ... ... ... ... ... ..- ... ... .-. ... ... .
```

Figure S2 (continued).
