# Supplementary material for: Cryptic Genetic Diversity within the Anopheles nili group of Malaria Vectors in the Equatorial Forest Area of Cameroon (Central Africa)
Source: PLoS One. 2013 Mar 14;8(3):e58862. doi: 10.1371/journal.pone.0058862 (PMC3597579; doi:10.1371/journal.pone.0058862)
Supplement: Table S1 — Estimates of null alleles frequencies per locus and per geographical population of An. nili s.l. from Cameroon. (DOCX) [file pone.0058862.s003.docx]

**Table S1:** Estimates of null alleles frequencies per locus and per geographical population of *An. nili* s.l. from Cameroon.

|  | *An. nili s.s.* | | | | |  | *An. carnevalei* |  | *An. ovengensis* |  | *An. somalicus* |
| --- | --- | --- | --- | --- | --- | --- | --- | --- | --- | --- | --- |
| Locus | Ako | Nkolbisson | Kentzou | Moloundou | Ekelemba |  | Afan- Essokyé |  | Nyabessan |  | Mbébé |
|  | 41 | 38 | 53 | 71 | 32 |  | 35 |  | 48 |  | 46 |
| 1D80 | 0.0547 | 0.0557 | 0.0344 | **0.0923** | 0.0000 |  | 0.0219 |  | **0.3276** |  | 0.0555 |
| 2Ateta | 0.0000 | 0.0400 | 0.0269 | **0.2419** | 0.0000 |  | 0.0287 |  | 0.0000 |  | 0.0788 |
| A154 | 0.0676 | 0.0000 | 0.1091 | **0.4165** | 0.0000 |  | **0.2955** |  | **0.2154** |  | - |
| B115 | 0.0000 | 0.0000 | 0.0000 | **0.1972** | 0.0000 |  | - |  | 0.0115 |  | - |
| 1F43 | **0.1539** | 0.0000 | 0.0653 | **0.1512** | - |  | - |  | - |  | - |
| 1G13 | 0.0000 | **0.1132** | - | - | - |  | 0.1362 |  | - |  | - |
| 2C157 | 0.0000 | 0.0267 | - | - | - |  | **0.3332** |  | - |  | - |
| F41 | 0.0370 | 0.0222 | 0.0000 | **0.1245** | - |  | 0.0856 |  | - |  | 0.0283 |
| 1A27 | 0.0406 | **0.1263** | 0.0436 | **0.1022** | **0.0000*** |  | - |  | 0.0738 |  | - |
| A14 | 0.0132 | **0.1284** | 0.0000 | **0.0873** | - |  | - |  | - |  | **0.2204** |
| F56 | **0.1618** | 0.0289 | 0.1054 | **0.1030** | - |  | **0.2030** |  | 0.0340 |  | 0.1005 |

Bolded: loci out of HWE after correction for multiple testing by the sequential Bonferroni procedure;

-: no amplification or no polymorphism detected;*: negative Fis value suggesting heterozygote excess.
