# Supplementary material for: Cryptic Genetic Diversity within the Anopheles nili group of Malaria Vectors in the Equatorial Forest Area of Cameroon (Central Africa)
Source: PLoS One. 2013 Mar 14;8(3):e58862. doi: 10.1371/journal.pone.0058862 (PMC3597579; doi:10.1371/journal.pone.0058862)
Supplement: Table S2 — Pairwise genetic distance estimates between An. nili s.l. D3 (above the diagonal) and ITS2 (below the diagonal) haplotypes from Cameroon. (DOCX) [file pone.0058862.s004.docx]

**Table S2:** Pairwise genetic distance estimates between *An. nili* s.l. D3 (above the diagonal) and ITS2 (below the diagonal) haplotypes from Cameroon.

|  | *An. nili s.s.* | | | | |  | *An. carnevalei* | *An. somalicus* | *An. ovengensis* |
| --- | --- | --- | --- | --- | --- | --- | --- | --- | --- |
|  | Type form* | Kentzou | Moloundou A | Moloundou B | Ekelemba |  |  |  |  |
| Type form* | - | 0.047 | 0.053 | 0.058 | 0.059 |  | 0.044 | 0.050 | 0.044 |
| Kentzou | 0.089 |  | 0.005 | 0.038 | 0.041 |  | 0.011 | 0.019 | 0.003 |
| Moloundou A | 0.098 | 0.018 |  | 0.038 | 0.041 |  | 0.019 | 0.022 | 0.011 |
| Moloundou B | 0.110 | 0.058 | 0.066 |  | 0.008 |  | 0.044 | 0.047 | 0.041 |
| Ekelemba | 0.129 | 0.078 | 0.075 | 0.031 |  |  | 0.047 | 0.050 | 0.044 |
| *An. carnevalei* | 0.112 | 0.130 | 0.140 | 0.162 | 0.186 |  |  | 0.008 | 0.013 |
| *An. somalicus* | 0.141 | 0.150 | 0.153 | 0.157 | 0.174 |  | 0.154 |  |  |
| *An. ovengensis* | 0.104 | 0.058 | 0.061 | 0.078 | 0.096 |  | 0.164 | 0.160 | - |

*: refers to *An. nili s.s.* from Ako and Nkolbisson.
