# Supplementary material for: Cryptic Genetic Diversity within the Anopheles nili group of Malaria Vectors in the Equatorial Forest Area of Cameroon (Central Africa)
Source: PLoS One. 2013 Mar 14;8(3):e58862. doi: 10.1371/journal.pone.0058862 (PMC3597579; doi:10.1371/journal.pone.0058862)
Supplement: Table S3 — Pairwise genetic distance estimates between An. nili s.l. ND4 (above the diagonal) and COII (below the diagonal) haplotypes from Cameroon. (DOCX) [file pone.0058862.s005.docx]

**Table S3:** Pairwise genetic distance estimates between *An. nili* s.l. *ND4* (above the diagonal) and *COII* (below the diagonal) haplotypes from Cameroon.

|  | *An. nili s.s.* | | | | |  | *An. carnevalei* | *An. somalicus* | *An. ovengensis* |
| --- | --- | --- | --- | --- | --- | --- | --- | --- | --- |
|  | Type form | Kentzou | Moloundou A | Moloundou B | Ekelemba |  |  |  |  |
| Type form* |  | 0.077 | 0.077 | 0.081 | 0.074 |  | 0.091 | 0.078 | 0.090 |
| Kentzou | 0.025 |  | 0.005 | 0.044 | 0.036 |  | 0.094 | 0.035 | 0.086 |
| Moloundou A | 0.025 | 0.004 |  | 0.043 | 0.035 |  | 0.094 | 0.035 | 0.087 |
| Moloundou B | 0.038 | 0.031 | 0.031 |  | 0.027 |  | 0.112 | 0.043 | 0.087 |
| Ekelemba | 0.041 | 0.035 | 0.035 | 0.016 |  |  | 0.098 | 0.035 | 0.085 |
| *An. carnevalei* | 0.047 | 0.042 | 0.042 | 0.050 | 0.049 |  |  | 0.109 | 0.095 |
| *An. somalicus* | 0.031 | 0.031 | 0.031 | 0.024 | 0.027 |  | 0.050 |  | 0.093 |
| *An. ovengensis* | 0.047 | 0.041 | 0.041 | 0.058 | 0.061 |  | 0.062 | 0.048 |  |

*: refers to *An. nili s.s.* from Ako and Nkolbisson.
